# Binders Used for the Manufacturing of Composite Materials by Liquid Composite Molding

**DOI:** 10.3390/polym14010087

**Published:** 2021-12-27

**Authors:** Ivan V. Terekhov, Evgeniy M. Chistyakov

**Affiliations:** 1 Federal State Unitary Enterprise, All-Russian Scientific Research Institute of Aviation Materials, National Research Center “Kurchatov Institute”, State Research Center of the Russian Federation, 17, Radio St., 105005 Moscow, Russia; 2 Department of Chemical Technology of Polymers, Mendeleev University of Chemical Technology of Russia, 9, Miusskaya sq., 125047 Moscow, Russia; ewgenijj@rambler.ru

**Keywords:** binders, liquid composite molding, preforms, automated fiber placement, composite materials

## Abstract

Binders, or tackifiers, have become widespread in the production of new composite materials by liquid composite molding (LCM) techniques due to their ability to stabilize preforms during laying-up and impregnation, as well as to improve fracture toughness of the obtained composites, which is very important in aviation, automotive, ship manufacturing, etc. Furthermore, they can be used in modern methods of automatic laying of dry fibers into preforms, which significantly reduces the labor cost of the manufacturing process. In this article, we review the existing research from the 1960s of the 20th century to the present days in the field of creation and properties of binders used to bond various layers of preforms in the manufacturing of composite materials by LCM methods to summarize and synthesize knowledge on these issues. Different binders based on epoxy, polyester, and a number of other resins compatible with the corresponding polymer matrices are considered in the article. The influence of binders on the preforming process, various properties of obtained preforms, including compaction, stability, and permeability, as well as the main characteristics of composite materials obtained by various LCM methods and the advantages and disadvantages of this technology have been also highlighted.

## 1. Introduction

**Figure 1:**
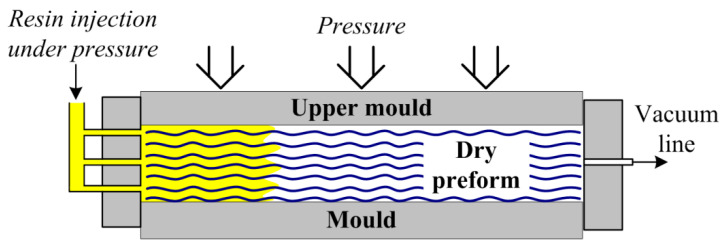
Scheme of the RTM process.

**Figure 2:**
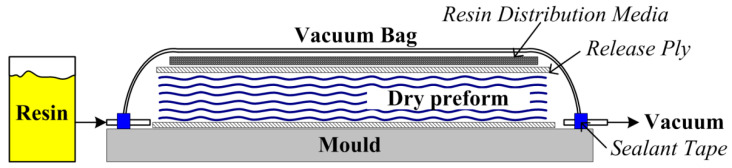
Scheme of the VARTM process.

Since the 1970s, the use of composite materials, especially fiber-reinforced polymer composites (FRPC), has become increasingly widespread in various industrial sectors, such as aerospace, automotive, energy, or sports industries. It happened due to their superior properties, particularly the light weight and high performance of mechanical properties compared to steel and other metals [1,2,3,4,5]. However, the further distribution of such materials to other sectors of industry is hindered by their high cost, since the initial autoclave/prepreg technology is highly labor intensive and requires a lot of energy for production of composites [6,7]. Furthermore, there are severe restrictions on the curvature of the obtained parts. In consequence of this several new methods of producing composites were developed, and the leading methods of reducing costs have become liquid composite molding (LCM) techniques such as resin transfer molding (RTM, Figure 1) or vacuum-assisted resin transfer molding (VARTM, Figure 2), also known as resin infusion (or sometimes vacuum-assisted resin infusion, VARI). The essence of these processes is the impregnation of a preform, made of dry fillers and laid out in the shape of the product, with a liquid matrix under the force of pressure (RTM) or with the help of vacuum, i.e., differences between ambient pressure and vacuum pressure (VARTM). It should be noted that sometimes VARTM is distinguished as a process in which additional pressure supported by vacuum is used for the injection, while VARI is distinguished as a process in which resin infusion is driven only by vacuum. Furthermore, RTM always refers to a process with two-sided rigid mold, while VARTM most often refers to a process with a single-sided rigid mold combined with a vacuum bag.

LCM processes can be significantly cheaper due to reduced labor cost and higher time and energy efficiency [7,8,9,10,11]. In addition, these methods make it possible to obtain products of complex shape in one cycle, which reduces the load of the equipment, and the resulting components are deprived of additional joints. However, there are a number of barriers to a wider use of this technique, e.g., the dimensional stability of performs during the impregnation, which can lead to smoothed corners, inhomogeneous thickness distribution, and as a result large resin rich zones and impregnation defects such as pores and dry regions [7,8,12]. High pressure during impregnation can lead to deformation and movement of a highly compliant textile laminate. Furthermore, the use of LCM in fabricating aerospace grade composites has been hindered by the difficulties in achieving mechanical performance and quality comparable to that of an autoclave/prepreg molded composite [13]. These problems can be solved by different methods, for example, tapes, staples, additional stitching with binder tows, etc., however, one of the most interesting options is the use of different binders [7,14,15].

**Figure 3:**
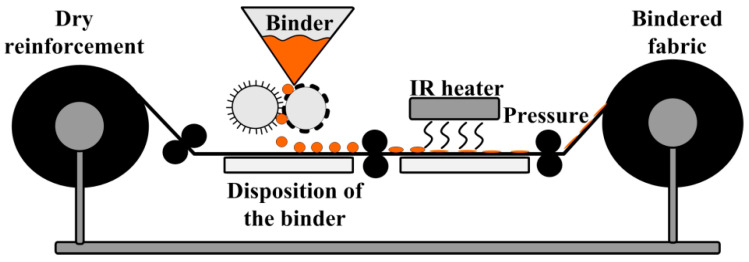
Scheme of typical binder application process.

Binders or tackifiers form an important part of the preforming process [7,12,13,14,15,16]. These are normally thermoplastic or thermoset resins or their blends that are solid at room temperature but melt easily on heating. The glass transition temperature (Tg) of the binder should be low enough so that the preform can be shaped without overheating or overcuring the binder [7,14,16]. At the same time the Tg should be high enough so that the preform can be handled and stored at room temperature. Usually, such binders can be in different forms, such as powder sprays, solvent sprays, veils, and emulsions. The form depends on the equipment that one have, but the most common in nowadays are powder binders or their combinations with veils and/or emulsions. The typical binder application process is shown in Figure 3. The choice of a binder is governed by several factors, such as compatibility with the matrix resin to be injected during the impregnation stage, operational effectiveness, process environment control, final product performance, and the preforming technique.

**Figure 4:**
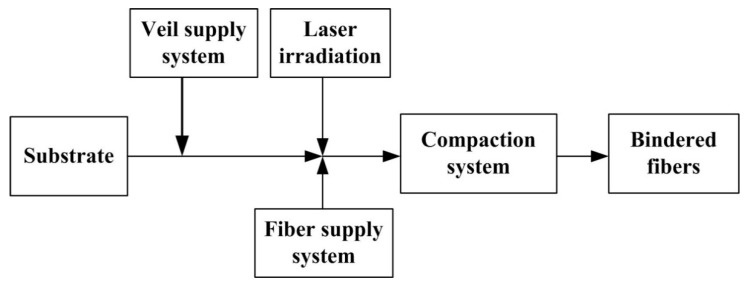
Scheme of the pulsed laser radiation for discrete binder application (adapted from [17]).

Moreover, new methods of applying binders to fibers in order to improve the quality and manufacturability of preforms were created recently [17,18]. For example, the use of pulsed laser radiation (Figure 4) can selectively heat and bind to discrete areas on a thermoplastic veil, used as a binder, to an incoming fiber tape material and a surface of a substrate. This contributes to a more precise control of the tacking of the veil to the fiber tape in predetermined locations.

In order to achieve sufficient consolidation of textile preforms a new approach was proposed, which suggests stabilizing a compliant preform through pointwise and highly controlled integration of a binder by the 3D printing of liquid resin into dry reinforcements, and its consolidation prior to liquid molding [12]. The same resin systems designed for LCM methods were used both for pinning and infusion. This helps to obtain a pattern that can be tailored to the requirements of infusion, draping, or tuning of composite properties. The printed binder resin created a stiff skeleton, securing material for resin infusion and curing.

In addition to the aforementioned applications, sometimes binders can carry another very important function, namely, to act as toughening agent, since thermoplastics can be added to the composite and reduce fragility of the cured polymer matrix [19,20,21]. Their use in the infusion processes as a toughening agent dissolved in the matrix is limited since they significantly increase viscosity of the different resins. A thermoplastic introduced as a binder can either dissolve during the impregnation of the matrix with the following reaction-induced phase separation, or remain completely insoluble during the impregnation and the curing reaction.

Another interesting application of the binders is their use in automatic laying out of the preform [2,22]. Thus, for example, a binder in the powder form applied to filler can give filler tapes which can stick together with each other under some pressure and heating. This significantly reduces labor costs of the manufacturing process and eliminates the influence of the human factor on the dimensional stability and quality of the preform.

Because of all the advantages mentioned above, such systems have become widespread in the world in the production of new composite materials. Recently, polymer binders are used for the production of roof frames, roofs, hoods, rear walls and other components of the sport and passenger cars [23,24,25] and for the production of MS-21 wings and wings-box [26]. In 1998, in the book Resin Transfer Molding for Aerospace Structures [7], binders, their types, as well as the process of applying binders to the fillers and the process of obtaining preforms from bindered fabrics were briefly reviewed. Binders were also mentioned in the review [2] devoted to automated preforming processes and some other works devoted to cost effective composite productions [27,28]. However, there have been no comprehensive reviews on this topic so far. Here, we try to summarize the knowledge about these materials, and describe their influence on the preforming process, preforms, and the obtained composite materials over 60 years, compiling, for the first time, the relevant literature on the topic. Moreover, the main issues that arise when creating and using such binder systems were highlighted, which will help engineers and researchers create such materials and research them. The sections of this article are devoted to the works on the binders, which can be used together with polyester, epoxy, and other types of liquid resins, as well as the literature considering impact of the binders on LCM technology and automatic preform layout processes.

## 2. Binders for Polyester Composites

**Figure 5:**
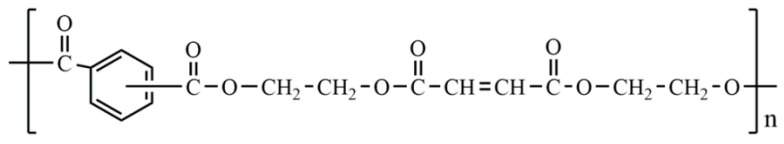
Chemical structure of typical polyester binder.

As the name implies, the LCM methods require that the resins used in the impregnation process should be liquid (the required viscosity should be less than 1 Pa·s or even less than 0.6 Pa·s). The first such compositions were polyester resins, since they can almost always be diluted with various liquid unsaturated monomers, for example styrene, with which the desired viscosity can be obtained. Therefore, in order to hold different glass mats together, the first binders were developed in the 1950s specifically for polyester resins (Figure 5) and often they were based on them [29,30]. Attention was also paid to melamine and phenolic binders, but polyester resins found greater use in LCM processes. Most of the described binders of this section are summarized in Table 1.

The main requirement for binders was that they must have sufficient strength to prevent disintegration of the glass fiber preform mats, susceptibility of the mat to uniform wet-out by the impregnating resin subsequently used, and that the obtained mats should be characterized by good drape and drawability so as to permit and facilitate their conformation to nonplanar shapes with or without mold pressure. An interesting fact is that before the invention of non-destructive testing methods, for example ultrasonic ones, there was a requirement for the cleanliness and whiteness of the mat binder, since the degree of impregnation of the resin and the quantity of defects was evaluated visually.

The first polyester resin compositions used as binder resins have different solubility rates in polyester and vinyl monomers [29,31]. The first task while creating binders was to control the solubility rate of the polyester binders in the vinyl monomer component since when applying different pressure on the system, different shear deformations arise. For example, in the low pressure operations, there is only a slight tendency for the fibrous material bonded by binder, so it is permissible for the polyester binder resin composition to dissolve moderately rapidly. In high-pressure operations, the tendency for the bonded material is greater and it is essential that the polyester binder holds the fibers firmly in place, and therefore, it should have a relatively slow solubility rate in the impregnated resin. Such control of the solubility rate was carried out by selecting the amount of poly-, di, and monofunctional monomers during the synthesis of polyester binders, which also determines the softening point of the binder. In addition, standard radical polymerization initiators, for example benzoyl peroxide, were used for partial crosslinking and to increase binder insolubility. The disadvantage of this partial crosslinking chain reaction is its unstable result, which can affect the dissolution rate.

An interesting idea was the use of a light curing binder in the manufacturing of glass fiber preforms for the production of composite materials by various LCM methods. D.T. Buckley suggested using a binder based on unsaturated monomers with two types of photoinitiators [32]. The first one was responsive to visible light and partly cross-linked the binder and provided a viscosity sufficient to hold glass fibers together. The second cationic-type photoinitiator was responsive to UV light and cross linked the preform in a given shape. This method reduces the cost of the manufacturing process, since it excludes the heating of the filler when applying the binder and when receiving the preform. However, photoinitiation strongly depends on the thickness of the resin layer, its shape, the amount and type of fillers, which restrains the widespread use of this method, since the binder can penetrate into the strand of fibers that does not transmit UV light. All these can lead to uneven curing. In addition, cationic polymerization is very sensitive to the impurities and can be easily inhibited.

The preforms with excellent shape shaping and stability, easy re-stretching and excellent compatibility with the vinyl resin matrix, excellent impact resistance characteristics and toughness can be obtained using binders based on powdered vinyl ester or acrylic resins with at least two unsaturated groups [33], preferably located at both ends of the chain, melting point in the range of 40 to 150 °C, viscosity at melting point in the range of 200 to 1000 Pa·s, glass transition temperature in the range of 35 to 120 °C, and average particle size of 20 to 500 μm. These binders can easily dissolve in the matrix resins during infusion and participate in the curing process. For example, bisphenol A-based vinyl ester resin VR-60 and an acrylic resin with epoxy groups PD-3402 can be used as such binders. Compared to polyamide Orgasol 1002 they have better peel strength and do not reduce flexural strength of the composites. The influence of these and other described binders on the properties of the preforms and obtained composites can be found in Table 2.

Since the 1990s, in view of the increasing spread of binder systems, academic science has actively begun to study them. By this time [37], thermoplastic binders were most widely used for polyester liquid matrices. Often low molecular weight thermoplastic polyesters soluble in the resins were used, since they allow faster wet-out of the preform. At the same time, such binders reduce the shrinkage of polyester resins, as thermoplastic additives are known modifiers used for these purposes [38].

For example, it was found that a preforming binder based on bisphenol A commercial thermoplastic polyester ATLAC 363E with fumarate groups after applying in an amount of 9 wt.% to the preform holds all the layers together and slightly decreases its thickness per layer [34]. The minimum thickness per layer with applied pressure was achieved with 3 wt.% of binder. Therefore, the highest fiber volume fraction may be expected from such materials under pressure applied during LCM processes. However, if the binder partially dissolves during the infusion of the reacting resin prior to the gelation, the binder may be detached from the fiber surface and its compacting effect may be lost, especially for VARTM processes, since the vacuum pressure is relatively low. It was found that the strain values along ply-lay-up loading direction of composites with compressive pressure were almost constant with the presence of the binder, while those along in-plane loading direction were a little bit increased (about 5%) by introduction of binder. This may be due to the fact that the fracture in in-plane loading is considerably related to the interlaminar properties that are modified by the thermoplastic binder. Furthermore, when 3 wt.% of the binder was added, both average compressive strength and modulus values (ASTM D-5528) increased slightly from around 490 MPa and 5 GPa to 550 MPa and 5.6 GPa and from 250 MPa and 7.9 GPa to 370 MPa and 9 GPa for ply-lay-up and in-plane loading direction, respectively. The further addition of the binder results in slight decreases of these properties. This may be associated with the optimum interlaminar strength and the highest fiber volume fraction. It was also observed by SEM that the extend of longitudinal splitting is greater in composites with binder. When studying the properties of a pure matrix mixed with a binder, it was found that mechanical properties of the model specimens are not significantly affected by the introduction of the binder. The viscosity of the resin lifts up from 1.135 Pa·s to 2.5 and 5.2 Pa·s initially by the addition of 6.5 and 12.85 wt.% of the powdered binder. The further increase of the viscosity during stirring was observed because of the partial dissolution of the binder within the matrix. The SEM investigation showed that there is no complete dissolution of the binder within the reacting resin system.

Moreover, this binder affects the mechanical properties of the E-glass/polyester composites [35]. It was found that the binder increases the peel strength of the preforms (Table 1) due to the improved adhesion by increased surface coverage of the fabric by the binder and reaches the highest values when the preform has almost full binder coverage on the glass fabric surface (approximately 9 wt.% of the binder). The flexural strength was decreased by about 20% due to the presence of 6 wt.% of the binder while flexural modulus reached 16.9 GPa, about 40% higher than those of the composite without binder. Mode I interlaminar fracture toughness of the composite laminates was significantly affected by the thermoplastic binder, i.e., 40% reduction by the presence of 3 wt.% binder. At the same time, the interlaminar shear strength (ILSS) was not significantly affected by the presence of the binder. The results of the ballistic test showed that as the concentration of the binder increases, a relatively less damage, i.e., less delamination, occurred within the back zones of the composites while no significant change was observed on the front zones. Unfortunately, both these studies do not show the effect of the ATLAC 363E binder on the glass transition temperature of the thermosetting resin or the composite, which is an important parameter that determines the operating temperature range of the material.

**Figure 6:**
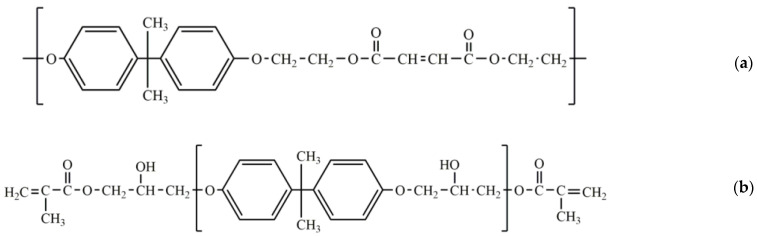
Chemical structure of ATLAC binder (**a**) and DERAKANE 411-C-50 vinylester resin (**b**).

In a comparative study of the effect of a catalyzed thermosetting powder binder PRETEX 110 and the ATLAC 363E binder on the properties of composite materials based on E-glass fiber and DERAKANE 411-C-50 vinyl ether resin (Figure 6), it was found that the two types of binders exhibit similar binder coverage and spread out and that the binder materials remain on top of the fiber mat surface [36].The powdered binder particles were first applied uniformly to the surface of individual fiber plies via a hand-held sifter type apparatus and then the mats were placed in a non-circulating oven at 65 °C for approximately 10 min in order to adhere the binder particles to the mat surface. It was noted that preforms constructed with the PRETEX binder were much stiffer and “board-like” than those fabricated with the ATLAC binder. The T-peel test showed that there is a much greater (15 times) interply adhesion for preforms constructed with the reactive epoxy binder than those with a low melting thermoplastic (Table 2). It is believed that the thermoplastic binder simply provides a mechanical bond between adjacent plies, whereas the reactive epoxy binder can provide an additional chemical adhesion. It was also found that specimens with unreacted PRETEX binder (processed at 65 °C for 30 min) exhibited very low adhesion, because at these conditions it softens but cannot flow and spread across the mat surface. Additionally, most of the binder applied at this temperature was poorly adhered to the fibers and could be easily brushed off. Predominant type of failure in both sets of specimens was an adhesive failure between the fiber and the binder. Additionally, the SEM images showed that ATLAC is a more brittle binder with the existence of fracture and cracking through the binder.

The composite laminates containing cured PRETEX binder had higher fiber volume fractions due to less preform springback (relaxation of the preform thickness) during infusion caused by the higher interply adhesion than the preforms with ATLAC binder has. Additionally, the ATLAC binder was soluble in the infusing resin, whereas once crosslinked PRETEX binder was not. The void contents lifted up from 1.78% to 2.11–4.64 and 1.63–3.00% by the addition of 3–9 wt.% of PRETEX and ATLAC 363E binders. Large voids concentrated in the interlaminar regions, and small voids within the fiber tows were found in the obtained composites. This suggests that the cured binder concentrated in the interlaminar regions inhibits the proper filling of the resin, thus creating interlaminar voids. Due to these voids the incorporation of both binder types into woven glass-reinforced vinyl ester composites results in lower interlaminar shear strengths. Composites with the PRETEX binder showed higher fracture toughness values compared to the control composite panel, whereas the composites made from preforms with the ATLAC binder showed 60% lower fracture toughness. It is believed that the PRETEX binder increased the G_IC_ propagation due to the high interply adhesion of the preform mentioned earlier. Additionally, if the ATLAC binder had not completely dissolved, it may act as a barrier between the matrix resin and fiber mat surface, inhibiting proper formation and chemical bonding at the fiber matrix interphase, and resulting in lower interply toughness. It was noted that the PRETEX binder mostly showed a cohesive failure between the individual fibers and the matrix (resin or binder), and the ATLAC binder showed a brittle cohesive-type failure, which suggests a tougher ductile type fracture. The study does not show the comparative effect of these binders on such important properties of composite materials as flexural or tensile strength, which substantially determine the performance of the product. In addition, the comparison of these different binders as reactive and non-reactive is difficult due to their different nature and their interactions with vinyl ether resin.

The ATLAC binder dissolves in the vinylester resin DERAKANE 411-C-50 via a “shrinking core” mechanism [39], in which the binder particle size gradually decreases over time, leaving a fairly homogeneous microstructure, and it affects the cure kinetics of the vinyl ester resin significantly delaying its curing. The delayed resin gel times and increased resin viscosity (for the sample containing 5 wt.% of binder they reach 37 min and 0.25 Pa·s, respectively, 50% and 90% higher than those of the matrix) could pose issues concerning the processing of composite laminates, and therefore, it may be necessary to modify the amount of curing agents to regulate curing times of the resin. It was showed that neither binder concentration nor extent of binder dissolution had any influence on the fracture toughness of the vinyl ester resin. The tensile strengths and strain to failures increased with higher binder loadings and greater extents of its dissolution from 29.79 ± 10.02 MPa and 0.91 ± 0.32% to 51.92 ± 9.72 MPa and 1.85 ± 0.50%, respectively, by the addition of 6 wt.% of the ATLAC binder (dissolution time was 24 h). Moreover, it improved the final glass transition temperature from 109.8 °C to 110.2 and 119.2 °C by the addition of 6 and 27 wt.% of the binder. The results showed that times in the range of 125–150 min were needed for the binder to reach nearly complete dissolution and mixing.

Thus, binders based on unsaturated esters are the most accessible, cheap, and adjustable in many characteristics, and especially they are suitable for the unsaturated resins. Other materials can also be used as binders, for example epoxy resins. However, they may not always be well combined with the reaction resin, and, sometimes, can reduce certain properties of the composite materials.

## 3. Binders for Epoxy Composites

The binder systems described in the previous section can be suitable not only for polyester or vinyl ester resins. Many binders, according to the manufacturers, are universal materials suitable for all or most types of resins. For example, Castro Composites suggests [40] their twill carbon fabric with epoxy binder to use with all type of thermosetting resins such as polyester, urethane-acrylate, vinyl ester, and epoxy. However, due to their different nature, it is often possible to find more suitable systems for certain types of resin. In this section, binder systems investigated for epoxy resins are described. Several of them are presented in Table 3.

The first work on binders for epoxy resins appeared around the same time [30], as the works on materials for polyester systems, however, due to the difficulty of obtaining low-viscosity epoxy resins for LCM processes at that time, these works became less common until the 1990s of the 20th century. The main problems that arose when using the already created and widespread binders for polyester resins with epoxy resins were poor compatibility and wet-out problems. Thus, the use of a glass fiber preform mat containing a polyester resin binder in producing a reinforced epoxy resin laminate results in a spongy, stiff, incompletely impregnated product which is unsuitable for use in many applications. A copolymer in the form of fibers containing about 40 percent of acrylonitrile and about 60 percent of vinyl chloride was proposed as a binder to solve these problems. This binder was added to the glass mat in an amount of about 4 wt.% or less and heated under pressure at a temperature of between 135 °C and 175 °C. This material has several advantages, for example it is possible to break up and recover both the glass and copolymeric fibers from mats not having acceptable strengths and to reform them into mats of acceptable quality. Moreover, such preform mats had better strength, they were lighter and loftier and had superior drape and drawability compared to the existing mats with emulsion-applied polyester resin binder. However, the main advantage was that they can be completely wet-out by the impregnating resin, particularly by the epoxy resins. Furthermore, composite materials based on them have sufficient flexural strength about 400 MPa, 43% higher than for composites based on existing mats.

A semi-crystalline diglycidyl ether of bisphenol A (DGEBA) solid binder (EPON^®^ RSS-1630) with substantial stiffness at room temperature was proposed to hold the fiber reinforcements together while infusion resin was a lightly crosslinked thermoset epoxy EPON^®^ Research Resin RSS-1623 FWRI with enhanced toughness [1]. This binder is miscible with the matrix system at elevated temperatures and it is preferred to make the homogeneous composite, but it inhibits the crosslinking reactions of the proposed matrix. It was observed that the proposed binder decreased the matrix Tg from 158 °C to 154 °C at low concentrations reaching an asymptotic value of 149 °C at higher concentration (more than 8 wt.%). However, the absolute decrease on Tg at all binder compositions was not of any significance and attributed to the binder interaction with the resin.

The study of the degradation temperature showed that the binder composition slightly increased it from 362 °C to 367 °C. This relation seemed to follow the rule of mixtures, where a mixture with a high content of thermostable resin had better thermal stability. Furthermore, the loss factor, which is indicative of the polymer network internal friction, increased with binder concentrations. After the postcuring, the 2% by weight binder also decreased the flexural strength from 152 to 140 MPa and slightly increase flexural modulus from 2.77 to 2.93 GPa, which was attributed to the fact that the epoxy binder interacts with the LXT matrix systems even at low binder concentrations by disrupting the final crucial crosslinking required for tough network structures.

In addition to partly cured thermosetting binders, non-catalyzed thermosets with sufficient properties can also be used for this purpose [41], for example a solid epoxy resin meltable above 40 °C, such as D.E.R.-662. The solid resin need not be identical to the injection resin but need only be compatible with it to prevent the pocket effect that occurs with thermoplastics. It can be applied to each layer by sprinkling or spraying in the amount of 1–15 wt.%. One of the advantages of using a non-catalyzed epoxy binder is both increased matrix and binder compatibility, and the ability to create 3D stabilized preforms of complex shape with a long storage time and the ability to relay preform. An additional benefit is that the compression and heating steps rigidified the preform in the desired shape and eliminated debulking as a problem in RTM molding. The effect of changing the ratio of the hardener to epoxy groups in such systems and the effect of the binder on the properties of the composite materials are not indicated in the patent.

Lopez and Pelletier [42] compared the compressive and short beam shear properties of composites prepared by powder and aqueous binder application techniques. The composite samples were made by using 5% by weight of D.E.R.-662 epoxy binder and the TACTIX123 epoxy resin as the matrix resin. They found that the shear strength and the compressive strength of the composite made from a powder-coated preform reduced from 61 MPa and 467 MPa by 21% and 19%, respectively, compared to the preform without any binder, while the shear strengths of the composite made by aqueous application increased by 2% and the compressive strength decreased only by 13%.

In addition to the uncured thermosetting binders, catalyzed materials also found use for these purposes. The increased compatibility of the binder and matrix was achieved using a binder on the same basis as the infusion resin [43]. The composite materials based on the precoated with a powdered version PS500 of the one-part liquid molding resin PR500 carbon tows had improved surface finishes and reduced void contents (1.4 vs. 5%) compared to composites without binder, which showed improved fibber wetting. The binder was applied by electrostatic powder fusion coating method. The matrix resin PS 500 based on the fluorene moiety, which enhances the glass Tg and providing high ductility and low moisture absorption, and has recommended resin injection temperature about 160 °C. It was also found that the fabric stiffness increased with the amount of powder coating on the preform, but the mechanical properties were not improved, probably because of defects inherent in the hand-woven towpreg fabric that was used. In this work, the powder resin was used not only like a traditional binder, but to obtain towpregs, where up to 40 wt.% of the resin were pre-impregnated in preform, which does not require additional impregnation.

The similar system based on PT 500 epoxy dry powder binder and PR500 RTM epoxy resin showed that the location of the binder would affect preform compaction and interlayer adhesion [13,44]. Binder was uniformly applied to one surface of two different carbon fabrics (As4-6k, 5 harness and IM7-12k, 5 harness), then the powder-coated fabric was placed in an oven or under an IR lamp at 100 °C for about one minute. Depending on the exposure time and the temperature during the preforming process the bindered preforms with cured binder either outside or inside the fiber tows were prepared. Different powder sizes were investigated and the size between 106 and 250 μm provided good preforming characteristics, particularly springback control, and was easy to handle. The preform with the binder outside the fiber tows showed better compressibility and springback control than the one with the binder inside the tows. The process with the constant pressure cycle showed a better springback control than the one with the pulse pressure cycle, but the difference is less if the location of the binder changed from inside the tows to outside the tows. If most of the binder stays inside the fiber tows, preform permeability may increase greatly because the binder inside the fiber tows shrinks the fiber tows and increases the space outside the tows. Moreover, it can be increased by high-preforming temperature (160 °C) and binder concentration in the range of 3–11% (at 11% the permeability is 2.34 times that of the unbindered fiber preform). Both flow visualization and microscopy of the composite cross-section showed that the presence of binder resulted in more voids (0.5–1.2% compared to 0.2% for composites without binder), but composites with binder inside the fiber tows had more voids and lower ILSS because such binder tends to cause microvoid formation inside the fiber tows, and the binder which stays outside the fiber tows tends to cause macrovoid formation outside the fiber tows, which can be easily purged by packing-and-bleeding. Thus, when packing and bleeding were applied during molding, the composites with binder outside the fiber tows showed no significant reduction in the ILSS for binder concentration up to 10%. The decrease in the ILSS from 75 to 50 MPa for materials with binder inside the fiber tows was attributed to void formation, because when specially obtained small specimens of composites without voids were investigated their ILSS was very close to the composites without adding binder. Adding too much binder causes some binder to stay outside the fiber tows and, consequently, the permeability of the fiber preform starts to decrease because it blocks the resin flow. It was found that the PT 500 binder needs to be completely cured in order to maintain its dimensional control ability; otherwise, the binder becomes soft or quickly dissolved in the hot resin and loses its ability for preform dimension control during molding. In this work the use of RTM and Seemann composites resin infusion molding process (SCRIMP) did not show any difference in the properties of composites. It was concluded that if the preform permeability is not a major concern, binder should be kept outside the fiber tows for applying for better preforming, lower void content, and better interlaminar shear strength. The influences of the binder on other important mechanical and thermal characteristics were not investigated in the work and also the processes occurring with the sizing agent at 160 °C, when the binder penetrated inside the fiber tows, were not taken into account.

To avoid possible deterioration of the matrix properties when using binders and anisotropy of the properties in the places where the binder was applied, it was proposed to use the same RTM6 epoxy resin as a binder during automatic preforming process [45]. In contrast to study with a pointwise binder 3D printing [12], in this work it was proposed to obtain a thin layer of resin on the fabric with spray nozzle and to use adhesive behavior of RTM6 at room temperature for cohesive bonding between two materials. After applying a controlled quantity of the resin material on some specific spots of a ply, pressure was applied to fix this ply with the component or other plies. Because the impregnation was carried out with the same resin material, the fixing spots on the cured component became invisible. However, since resin does not cure in this method, it cannot provide stiff preforms.

**Figure 7:**
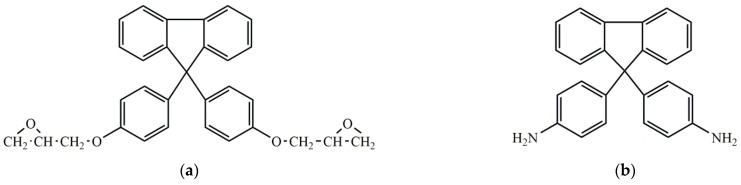
Chemical structure of a diglycidyl ether of a 9.9-bis(hydroxyphenyl)fluorene (**a**), and a 9,9-bis(aminophenyl)fluorene curing agent (**b**).

The powdered binder based on aromatic polyepoxides, a fluorene-containing epoxide based on diglycidyl ether of a 9.9-bis(hydroxyphenyl)fluorene, and a 9,9-bis(aminophenyl)fluorenecuring agent (Figure 7) can be used with different epoxy RTM resins, PR-500 in particular [66], and it is uniquely compatible with them, which helps to provide a uniform distribution of the binder resin in the ultimate composite article. Such powdered binder with the preferable particle size between about 40 and 80 μm can be applied by melt extruding a dry blend of these ingredients without advancing (i.e., partially curing or B-staging) them before further use and by different other methods. The uncured binder resin displays a glass transition temperature of greater than 40–65 °C, along with a minimum viscosity of less than about 100–10,000 Pa·s. All these advantages attributed to interesting properties of fluorene components of the binder, e.g., high glass transition temperature in uncured form. These binders did not affect the Tg, tensile modulus, compression strength, and in-plane shear strength of the cured PR500 epoxy resin, which indicate their mutual excellent compatibility.

Two binders modified with polyamide 6 particles (Orgasol 1002 D Nat, 22 µm) were proposed to obtain improved interlayer toughness of resin transfer molding composites [20]. The first was liquid binder for spray application, based on DGEBA (Epon 1007F), and the second was powder binder PT500. Both binders contained 33 wt.% of polyamide particles and they were applied manually only onto one side of the fabric with 3.8 wt.% binder-resin loading, which corresponded to 1.9 wt.% particle loading (Table 3). It was found that the interlayer-toughening concept based on addition of thermoplastic toughening agents such as polyamide, polysulfone, etc., to the epoxy resin and initially developed for prepreg materials is applicable to RTM processes. However, they can improve some properties and decrease others (Table 4).

The powder binder was less evenly distributed on the textile surface than the spray binder. It was found that the particles were not washed out during injection and remained in the interlayer where they were initially applied. The non-uniform distribution obtained by the powder binder resulted in a less homogenous interlayer in the laminate structures. Mode I interlaminar fracture toughness was slightly decreased from 300 J/m^2^ to 250–270 J/m^2^ using the modified binders. The better particle distribution in spray binder preforms showed 30% improvements in mode II interlaminar fracture toughness up to 2.1–2.2 kJ/m^2^ and no significant change in the ILSS. The particulate modified commercial powder binder showed a slightly lower performance increase in mode II and slight reductions in the ILSS partially due to the ununiformly layered laminate structure. The size of thermoplastic particles can significantly affect the interlaminar fracture toughness of the composite material, however, the authors did not conduct these studies.

In order to abandon the use of a polyamide dispersion in epoxy resin and to increase the processability of the polyamide-containing binders, a mixture of amorphous polyamides with a glass transition temperature of 140 °C or higher and comprising a dicyclohexylmethane unit, with different toluenesulfonamides was proposed as suitable replacement [49]. Such binders due to the high compatibility of polyamides and toluenesulfonamides had reduced glass transition temperature of the binder composition in the range of 40 to 90 °C, which means that such binders were easier to handle. The toluenesulfonamides can act as a curing agent for the epoxy resins during infusion and the curing process [67], but because of their little concentration these binders had little effect on neat epoxy resins. For example, when the mixture of 10 parts by mass of the binder composition with 100 parts by mass of an epoxy resin was stirred at a temperature of 180 °C for 1 h, the viscosity of the filtrate obtained by filtering the solid particles of polyamides from the mixture was in most cases four times or less the viscosity of the epoxy resin not yet mixed. The composite materials based on preforms obtained with the help of such binders also had improved from 190–250 (6.76 J/mm) to 221–290 MPa compression after impact (CAI) strengths. The other thermal and mechanical properties of obtained composite materials were not investigated.

**Figure 8:**
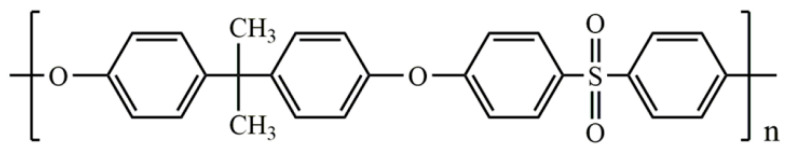
Chemical structure of polysulfone.

An alternative way of introducing a thermoplastic agent into a binder is to use polysulfones or phenoxies (Figure 8) soluble in epoxy resins [46,47,48]. So, for example, binders based on one or more multifunctional epoxy resins and polyarylsulphones in the form of water emulsions with particle size about 0.5–5 µm, viscosity 0.3–0.6 Pa·s, and solid content 55–60% had several advantages: these compositions are environmentally friendly; they can homogeneously coat fibrous tows in fabrics, thereby enhancing the fiber tows’ integrity; they provide adequate levels of adhesion/compatibility to conventional infusion epoxy-based resin matrix and provide excellent drape ability with few creases and shrinkage less than 1%; they are able to minimize/eliminate the variability in bond-ability normally observed in powder-coated textiles; they have limited or no impact on the thermo-mechanical performance of the composite part produced from a fibrous preform that has been treated with the liquid binder and impregnated with Prism EP 2400 epoxy resin. In some samples the compression strength was reduced from 1226 to 1125 MPa. However, in some cases such binders can improve compression strength up to 1298 MPa and increase inter-laminar shear strength (Table 4). Moreover, such binder compositions can be formulated to have a solid content and a viscosity level that enable the application by dip-coating or spraying at ambient temperature. Preforms based on bisphenol A phenoxy-modified epoxy resins with a molecular weight of 30,000 to 65,000 Dalton improved permeability and compatibility with epoxy resins and they can reduce injection time and improve fiber wet out without change on Tg. The authors have not shown the influence of such binders on other properties of composite materials.

The opposite effect can be observed when using traditional for polyester resins thermoplastic polyester binders [19]. Although 5–6 wt.% of the powdered thermoplastic polyester binder with the average particle size of about 250 µm provides full coverage of the glass fabric surface and gives good peel strength of the preforms about 0.39–0.78 N/cm, the use of such binders can negatively affect the glass transition temperature and fracture toughness of the epoxy composite material. For example, the interlaminar shear strength, Mode I interlaminar fracture toughness and glass transition temperature of the S2-glass/SC-15 epoxy resin composite system with 2.6 wt.% of polyester thermoplastic binder reach 30.95 MPa, 617 J/m^2^, and 95 °C, respectively, 25%, 60%, and 6 °C lower, than material without binder. Furthermore, it was found that the polyester binder had limited dissolution in the composite panels based on epoxy resins which were processed under standard cure cycle. The study does not investigate the effect of larger amounts of the binder on composite properties and does not describe the method of its application.

At the same time if some precautions are taken in mind, interlaminar toughness of epoxy composites can be increased without negatively affecting other properties by coating the reinforcing plies with a bisphenol A-based thermoplastic polyester based on poly(4,4′-dipropoxy-2,2′-diphenylpropane fumarate) [50,51]. It was shown that by a proper selection of the amount of applied binder, the detrimental effects can be avoided and the mechanical properties can even be increased (Table 4). DSC experiments showed that the polyester is miscible in the epoxy resin at low amounts (less than 9 wt.%) without forming polyester-rich globules. At higher polyester loadings, a two phase morphology forms which consists of polyester-rich globules in epoxy. SENB (single-edge notched bending) experiments indicated that polyester-rich globules had a detrimental effect on the fracture toughness of the bulk epoxy matrix resin. It was also showed that the polyester added to an epoxy resin in low amounts acted as a plasticizer for the cured bulk epoxy resin and slightly decreased its Tg from 88 to 84 °C and increased its bulk fracture toughness by 30% when 4.8 wt.% of binder was added. The further addition of the binder decreases Tg to 72–76 °C. 

Moreover it returns bulk fracture toughness to the original values. This could be due to polyester molecules blocking crosslinking sites leading to a reduction in reaction enthalpy. Furthermore, at high polyester loadings the diffusion of epoxy and amine curing agent molecules can be hindered which results in an additional decrease in crosslink density. At higher polyester loadings, the immiscibility of the polyester in the epoxy resin results in the formation of a brittle polyester-rich second phase. Polyester-modified glass/epoxy laminates showed increased toughness of the epoxy matrix. For example, polyester loading of around 3 wt.% led to a 60% increased Mode I interlaminar fracture toughness of the laminates both in initiation (from 410 to 650 J/m^2^) and propagation (from 610 to 1000 J/m^2^), without negatively affecting flexural stiffness and strength of the laminates. At higher polyester loadings up to 6 wt%, the interlaminar fracture toughness stays more or less the same. The increase in interlaminar fracture toughness can be explained by the locally increased failure strain of the epoxy resin in the interlaminar regions due to the plasticizing effect of the polyester on the resin and by the absence of large quantities of polyester-rich phase. Moreover, it was found that there was no significant effect of the addition of 3 wt.% of such binder on the flexural strength and Tg of the composite. It should be noted that the chemical structure of the binder presented in the article is not correct.

Interesting materials which can be used as binders are different thermoplastic veils, or non-woven materials [52]. Such veils can be melt-bonded to the unidirectional fabric and provide good preforming properties. The best advantage of these materials is their toughening properties. Several of the veil materials, for example polyamide PA1008 and ternary elastomer VI6010 increased CAI performance of composites based on the TV-15 and VR56–19 epoxy resins and T700 carbon fibers (Table 4). The value depended on the interlayer areal weight. Moreover, they improved room-temperature and dry mechanical properties of the composites. In contrast, PE2900 polyester veils dramatically reduced CAI performance and reduced mechanical performance with increasing veil areal weight, which correlates with the above data about polyester powder binder with epoxy resin. Moreover, the three studied veil adversely affected the hot/wet properties when added in increasing amounts due to their low-melt formulation and their hygroscopicity. Newly developed improved polyamide veils yielded in improved hot/wet performance, but not all of them led to good CAI strength. Veils based on thermoplastic polyurethanes (TPU) and their mixtures with polyamides did not affect the composite properties. This is believed to be due to the low modulus of the TPU. This suggests that a veil material must have a modulus that is not significantly lower than that of the underlying resin.

Veil, based on low-melting temperature and matrix soluble phenoxy, was proposed as binders for epoxy resin RTM6 [53]. The preform based on such phenoxy non-woven PKHP200 was compared with preforms stitched by polyamide (Grilon K 140) or phenoxy (Grilon MS150) yarns and preform based on non-woven polyamide PA1541. The two non-woven binders were incorporated between either each biaxial layer or every second biaxial non-crimped carbon fiber (NCF) layer. When using different yarns a good handling of the dry NCF preforms was achieved. The obtained preforms had an enhanced stiffness and stability, which was even further improved when adding the polyamide and phenoxy non-wovens as additional binder. The cross-sectional computer tomography showed the reinforcement fibers displaced from their original alignment in the vicinity of the stitching yarn. It was found that stitching and bindering using the employed polyamide materials significantly degraded the dry and the hot-wet ILSS properties of the composite materials from 68–75 to 55–68 MPa. In contrast, the phenoxy-stitched and bindered composites revealed only a minor reduction of the shear strength performance as compared to the non-stitched and non-bindered NCF reference in both cases. The inferior dry ILSS properties of the polyamide-containing composites indicated either a poor polyamide-reinforcing-fiber interface or a basic inability of this polyamide to bear and transmit shear forces.

The G_IC_ properties (fracture toughness) of the reference material were dramatically improved by using both the polyamide and the phenoxy non-woven binders. The effect was more distinct for the polyamide-bindered composite material (Table 4). A phenoxy-modified laminate without a non-woven placed in the crack plane exhibits no improvement as compared to the reference system. The superior fracture (mode I) properties of the bindered composites are due to significant plastic deformation in the fracture zone caused by the polyamide or phenoxy typical ductile fracture behavior. The G_IIC_ properties of the reference material were improved from 1100 to 1600 J/m^2^ only by using the phenoxy non-woven binder. The G_IIC_ of composite based on preform stitched by phenoxy yarns reached 1400 J/m^2^. The explanation for the observed improved G_IIC_ performance of the phenoxy-modified composites can be attributed to matrix hackling. When using binders none of the composites showed significantly improved CAI properties and no clear change in delamination size could be deduced from the ultrasonic c-scan data. Moreover, they did not affect the compression modulus of the non-crimped fabric composites, but they reduced the compression strength of the laminates by app. 8% from 870 MPa. The influence of such veils and yarns on other mechanical and thermal properties has not been shown.

In addition to the mentioned thermoplastics, for the production of such binder veils polyimide, polyamide-imide, polyester, polybutadiene, polyurethane, polypropylene, polyetherimide, polysulfone, polyethersulfone, polyphenylsulfone, polyphenylene sulfide, polyetherketone, polyetheretherketone, polyarylamide, polyketone, polyphthalamide, polybutylene terephthalate, polyethylene terephthalate, polyester-polyarylate, polyaramid, polybenzoxazole and viscose fibers can be used [54]. There are also works where powder binders or water borne dispersions based on different thermoplastics (polyamides, polyhydroxyether, polyphthalamides, polyurethane, their copolymer or their reaction products, Phenoxy 85, Phenoxy 95 and copolyamidesCoPA 130, CoPA 180 and CoPA 300, polyethersulfones and others), epoxies (for example a 7P1610 epoxy powder or a thermoplastic modified, epoxy-based binder Cycom R. 7720) or their mixtures and different thermoplastic veils are proposed to use together to form veiled tape that is resistant to delamination and has good drape ability, anti-fraying behavior, and low shrinkage [55,56]. Thus, for example, the required shear force was increased from 100 to 300 N with the addition of 5 wt.% of 7P1610 epoxy powder binder inside the fiber tows of the composite material based on HT40F13 carbon yarns and 1R8D06 polyamide veil. Binder applied only to the surface did not show any change in shear forces. In addition to thermoplastic veils the thermoplastic nylon fiber in the form of thermal bonding mesh layers can also be of interest [57]. When 2.08 wt.% of such binder was used in the composite material, its ILSS was improved from 48.1 to 50.6 MPa. Further addition of the binder caused a decrease in ILSS of composites down to 38 MPa at 10.42 wt.% binder content. Despite the great advantages, veils also have some drawbacks, namely, their high cost and complexity of their production, as well as the complexity of their use in the manufacture of complex-shaped composites, since they limit the uniform application of all plies.

Such thermoplastic binders can also be used only in the powdered form [58]. They can provide preforms with high fiber cohesion, sufficient enough to hold the fibers, at room temperature, in the desired shape and position but small enough to leave the resulting preform porous. For example, Phenoxy 85 and Phenoxy 95 (phenoxy resins with medium and high molecular weight), CoPA 130, CoPA 180, and CoPA 300 (copolyamides with different glass transition temperatures) applied to Toho Tenax^®^ J IMS60 E13 fibers in an amount of 18 g/m^2^ can improve CAI of composite materials obtained with RTM 6 and Cycom 890 RTM epoxy resins (Table 4). The best improvement showed CoPA 130 and CoPA 180 binders. Less amounts of binders had less impact on CAI properties. Influence on other properties was not indicated in the work.

The influence of powdered binders, such as polyhydroxyether/phenoxy (Grilon MS), polyester (KE-60), co-polyamide (K-140), co-polyester (D 2433E), and also an epoxy binder (Epikote 05390) on properties of epoxy resin-based composite materials used in the manufacturing of wind turbine rotor blades was carried out by German Aerospace Centre [14,59]. The epoxy resin Airstone 880E with the corresponding amine hardener Airstone 886H was used as the polymer matrix and an unidirectional E-glass fabric U-E-1200 g/m–1300 mm with a weft content of 5 wt.% was used as fiber semi-fabric. The binders were sprinkled onto the lower glass fabric surface using a sieve. It was confirmed that binders have to be chosen carefully to meet the requirements of the preform process (high interply adhesion) and the resin infusion process (low solubility within resin and slight effect on viscosity) as well as to reach the needed overall mechanical properties of glass composites. The two binders KE-60 and Epikote 05390 were found to be critically soluble in the matrix. Extreme increases in viscosity from 0.1 to more than 0.5 Pa·s in 60 min and complete solubility at room temperature were observed, respectively. The three remaining binders were classified as strongly soluble (Grilon MS), partially soluble (D 2433E), and nonsoluble (K-140). In the case of resin-binder plates with soluble phenoxy-binder Grilon MS, independent of binder content, the tensile strength, modulus, and fracture strain did not differ from the reference and the glass transition temperature decreased only slightly. The binders K-140 and D 2433E result in a linear drop in tensile strength and modulus from 60.3 MPa and 3 GPa to 54.7 MPa and 2.56 GPa respectively with increasing the binder content, and in case of partially soluble polyester binder D 2433E this effect was slightly stronger. It was concluded that the development of resin-binder interfaces should be avoided to reduce any degrading morphological effects, which increase at higher binder contents. It can be obtained by using strongly soluble or partially soluble binders.

The peeling tests showed that the binder layer of K-140 shows the lowest peel strength (4.8 N/cm) and exhibits gaps at the lowest binder loading (24 g/m^2^), which were closed at higher binder loadings, corresponding with an increase in peel strength (7 N/cm). Grilon MS and D 2433E binders, due to their better wettability, showed maximum peel strength (10.5–11 N/cm) starting from the lowest loading, and due to a gapless binder layer, an increase in binder loading increases interlayer thickness but does not enhance binder-fiber interaction. Furthermore, all investigated binders showed only small intraply migration during loading and preforming.

Mechanical tests of composites for tension, compression, and shear strengths showed the same trend for the different binders and binder contents (1 and 3 wt.%) with varying intensity [59]. It was also showed that fracture occurs directly without the binder; with the binder, final failure is delayed (slightly for Grilon MS and strongly for K-140 and D 2433E). This phenomenon confirms the influence of the binder’s solubility. The non-soluble and partially soluble binders K-140 and D 2433E form a clear binder–matrix interface which acts as a potential weak spot where cracks can be initiated. However, these weak spots can also deflect existing cracks along the interface changing their mode from crack opening to in-plane and out-of-plane shear, which results in delayed failure and more pseudo-plasticizing deformation. The strongly soluble Grilon MS binder showed the best performance that was comparable to the binderless reference. It only slightly reduced the tensile strength and modulus and compression strength and modulus in longitudinal direction and improved the compression strength and modulus in transversal direction from 135 MPa and 13.9 GPa to 147–155 MPa and 14.6–15.2 GPa, respectively. The K-140 and D 2433E binders reduce the performance especially for the high binder content. In contrast to the bulk partially soluble epoxy resin binder D 2433E had a more critical influence on the composite than the non-soluble binder K-140. Thus, not only the binders solubility influenced the composite mechanical performance. Since the drop in mechanical properties was moderate for low binder contents, it was concluded that the binder content should be kept low as long as preform properties are still sufficient. However, higher binder contents may lead to more efficient toughening (e.g., fracture toughness, CAI, etc.,) in the composite when chosen carefully.

Thermoplastic thin (0.05 mm) film, used in the hot-press tackification process, showed the enhancement of the mechanical properties of the cured laminates based on Araldite LY 1564SP epoxy resin and a Hardener XB 3486 curing agent [60]. The hot-press tackifying process was conducted by placing the thermoplastic film between two fabric layers and then, heating it at 130 °C for 30 min in an autoclave at pressures of 0.1 and 0.6 MPa. The in-plane permeability of the modified preforms treated at 0.1 and 0.6 MPa were 1.4 × 10^−11^ and 1.9 × 10^−11^ m^2^, respectively, slightly less than the permeability of the unmodified preform (2.5 × 10^−11^ m^2^). It was found that the modified laminates at 0.1 MPa had a better interfacial feature. Modified composites obtained by VARTM had improved thickness uniformity and increased the tensile strength and compressive strength, reaching 1750–2160 MPa and 190–400 MPa, respectively, 600–1000 MPa and 75–290 MPa higher than those of the composite materials without binder. A similar modified polyether sulfone thermoplastic film (L-120), treated at 0.1, 0.3, and 0.6 MPa, also increased the tensile strength and modulus of the modified laminates based on unidirectional T700 carbon fiber fabrics and a low-viscosity EA 170 matrix with hardener XB 3486 from 1465 to 1712–2070 MPa and from 121 to 124–137 GPa, respectively [61]. The modified laminates at 0.3 MPa have the best distribution between the fabrics and the thermoplastic films, which corresponds to the best mechanical properties of the laminates. The unmodified samples showed a brittle fracture with a smoother fiber surface, compared to the modified samples. Unfortunately, the authors did not provide details about the thermoplastics used in the work, as well as their influence on the fracture toughness.

In order to achieve a sufficient tackiness of bindered preform for applications in which yarns are laid over each other at specific angles, a two-component resin binder was proposed wherein these resins were applied on fibers separately [62]. Such systems consisted of a water dispersion of solid and liquid epoxy resins modified with an aromatic polyhydroxy ether and similar powder or another water dispersion of solid epoxy resins with a melting temperature in the range from 80 to 150 °C. The obtained preforms based on these binder systems were very dimensionally stable due to the high adhesive strength of the pre-impregnated yarns used and could be handled problem-free for further processing (infusion with RTM-6 epoxy resin). A force of 429–678 N was required to separate such bindered yarns adhering to each other, resulting in a shear tensile strength of 3.68–4.44 N/mm^2^. It was shown that such mechanical characteristics as ILSS, the compressive strength, and the compressive modulus of the composite laminate produced with the pre-impregnated with binder yarns were at the same level as corresponding characteristics of a laminate based on standard carbon fiber yarns (Tenax HTS40 F 13 12 K 800 tex), even though the concentration and the composition of the resin differed significantly from the standard material.

Interesting idea was the use of multiwall carbon nanotubes (MWCNTs) in epoxy binders for manufacturing multi-scale composites with improved interlaminar fracture resistance [63]. The modified compositions were based on the 5-harness satin weave and the mixture (100:35) of Araldite LY 564 (DGEBA/butane diol-diglycidyl ether) and Aradur 2954 (2,2′-dimethyl-4,4′-methylenebis(cyclohexylamine)) used as an infusion resin. An aqueous dispersion of a solid DGEBA epoxy resin, EPI-REZ 3522-W-60 was used for production of non-curable and curable epoxy binders with a 1 wt.% dispersion of NC7000 industrial grade MWCNTs. A mixture of dicyandiamide and 2-methylimidazole was used as a curing agent for curable binder. The weight of binders after drying was around 6.5 wt.%. The uncured binder and matrix epoxy system formed a multiphase matrix due to reaction-induced phase separation which increased values of G_IC_-Prop and G_IIC_-Prop ILFT from 0.47 and 1.38 kJ/m^2^ to 0.63 and 1.74 kJ/m^2^ for neat binder (34 and 18% increase) and to 0.99 and 2.33 kJ/m^2^ for binder with 1 wt.% MWCNTs (110 and 57% increase), respectively, but also caused significant plasticization of the matrix and reduced the main Tg from 140 °C by up to 45 °C. This plasticization effect was partially reduced by using the curable binders. Neat curable binder also increased G*IC*-Prop to 0.88 kJ/m^2^ (by 87%) and G_IIC_-Prop to 2.33 kJ/m^2^ (by 40%). The highest increases in ILFT to 1.54 kJ/m^2^ (by 234%) in G_IC_-Prop and to 3.04 kJ/m^2^ (by 106%) in G_IIC_-Prop gave the cured binder with 1 wt.% MWCNTs. The addition of MWCNTs also inhibited the plasticization of the matrix, reducing the drop in Tg by approximately 25 °C.

**Figure 9:**
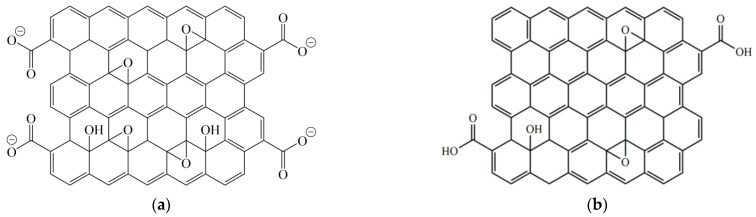
Proposed chemical structure of the graphene oxide (**a**) and partially reduced graphene oxide (**b**).

Other carbon nanomaterials also can be used as nanocarbon binders [64,65]. For example, graphene oxide can be embedded to the surface of carbon fabric by anodic electrophoretic deposition process and act as an effective binder, small amount (0.0003 wt.%) of which can improve the binding force of fiber bundle by 105% to 11.65 N. It was less than for an ordinary polymer binder (22–23 N), but it was enough to bind fibers and it did not decrease permeability of fiber preform in contrast to polymer type binder. Furthermore, it increased the interlaminar shear strength of composite materials based on plain woven carbon fabric TR-30 and epoxy resin PRISM EP 2400 by 13.6% to 79 MPa. The partially reduced graphene oxide (Figure 9) also can increase ILSS from 63 to 69–71 MPa and effectively enhanced electrical conductivity of the carbon fiber composite from 10 to 12–13 S/cm in-plane and from 0.004 to 0.02 S/cm through the thickness. The increase in electrical conductivity is owing to the formation of electrically conductive networks of the coated pRGO sheets within and between the carbon fibers.

Binders for composite materials based on epoxies have advantages when used with epoxy liquid RTM resins. They are well compatible with them, and also their properties can be well controlled by changing their composition. At the same time, binders modified with thermoplastics or consisting only of them are of great interest, since they increase fracture toughness of epoxy resins. However, as in the case of unsaturated resins, they can reduce some properties when used with poorly compatible matrices.

## 4. Binders for Other Matrices

**Figure 10:**
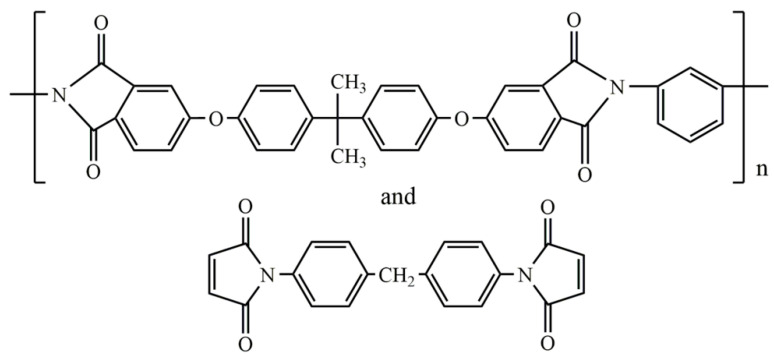
Chemical structure of two-component binder based on polyetherimide and bismaleimide resin.

It is most often recommended to choose a binder of the same nature as the infusion matrix used to impregnate the preform [41,68,69,70]. For example, a thermoplastic polyamide 12 powder binder is recommended for the thermoplastic resin transfer molding of *ε*-caprolactam, which can be anionically polymerized into PA6. Furthermore, a bismaleimide resin Cytec 5250-4 modified with polyetherimide Ultem 1000 (25 wt.%) can be used as a binder for the same resin and improve the G_IC_ of the obtained 7781-style glass fabric composite from 269 to 420 J/m^2^, and a binder modified with 40 wt.% of polyethersulfone Victrex 5003P improves it to 448 J/m^2^. However, as can be seen from the aforementioned information, some binders can find application with different resins. In this regard, some commercially available binders for various LCM technologies can be used with various polymer matrices, and if they are miscible, soluble, or mutually compatible with each other it is the great advantage [71]. That is why, due to the mutual compatibility between epoxy resins and bismaleimides and benzoxazines and the possibility of their reaction with each other, in some cases, epoxy-based binders can be used for bismaleimide and benzoxazine matrices and vice versa [72]. For example, for bismaleimide resins or epoxy resins with amine hardeners, a binder based on polyetherimide and bismaleimide (Figure 10) can be suitable. Binder in the form of water suspension based on bisphenol-A epoxy resin, dicyandiamide as a curing agent, polyacrylamide as a thickener, and imidazole catalyst was proposed for use with such compatible matrices as vinyl ester, polyester, phenolic thermoplastics, and most preferably, urethane polymers [73]. Similar epoxy binder can even be used with novel thermoplastic resin system [74,75].

Another example is a preform with two different binders in the form of aqueous dispersion, which was developed for polyamides, copolyamides, polyurethanes, and epoxy polymer matrices [76]. The carbon yarns were pre-impregnated with the first binder (15 wt.% of solid phase), which consisted of urethane resins produced from a reaction of a DGEBA resin, an aromatic polyisocyanate, and a polyalkylene glycol; an oxyalkylatedbisphenol A resin and aromatic polyhydroxy ether with an average molecular weight in a range of from 4000 to 5000 g/mol. The second binder resin composition was on the bundle on the outer side in the form of particles or drops adhering to the reinforcing fibers and comprised a DGEBA resin, an aromatic polyhydroxy ether, or other thermoplastic polymer (polyamide, polyethylene, ethylene copolymer, thermoplastic polyurethane resin, or their combination). The components and their concentrations were selected in order to obtain a binder, on the basis of which it would be possible to obtain preforms with high dimensional stability, compatible with different matrices, however, the principle of choosing a thermoplastic polymer is not very clear. Nonetheless, the authors specified that both binder compositions had sufficient compatibility for use with the matrices indicated above. At the same time, only the ILSS of the composite material obtained with RTM6 epoxy resin was shown, and it remained at the level of the material without binder.

Epoxy preforming powdered binder EPIKOTE Resin 05390 with melting point of 90 °C was found suitable when used with catalyzed cyclic butylene terephthalate (CBT) oligomers [21,77,78], the attractive oligomer systems with unique properties, such as low melt viscosity (0.03 Pa s at 190 °C) and the rapid polymerization to polybutyleneterephthalate (PBT) with virtually no heat generation and without the evolution of low-molecular-weight byproducts. The reason of using epoxy binder was that the possible reaction between PBT and epoxy resins can lead to a tougher PBT. It was found that the presence of binder can impede the crystallization of pCBT (polycyclobutyleneterephthalate), which is the main approach applied for toughening of isothermally produced pCBT matrix. The crystallization temperature and the crystallinity of pCBT polymer are both found to decrease with increased filling fraction of preforming binder. The presence of 2 wt.% of preforming binder leads to a reduced melting enthalpy from 38.15 J/g to 30.72 J/g. Further addition of preforming binder up to 6 wt.% has no further significant influence on the melting transition of CBT oligomers. The crystallization temperature was shifted from 199.85 °C to 185.74 °C and the crystallization enthalpy decreases from 52.63 J/g to 42.39 J/g with the filling fraction of preforming binder increases from 0 wt.% to 6 wt.%. It was also indicated that the processing time of the catalyzed oligomers during isothermal polymerization can be prolonged due to the presence of preforming epoxy binder. The manufactured textile-reinforced pCBT composites with 2 wt.% of binder showed a significantly increased flexural strain at break from 1.59% to 2.75% and flexural strength from 420 MPa to 711 MPa, without impairing other mechanical properties such as flexural modulus, compared to the composites produced without binder. Further addition of the binder has negative effect on these properties.

The other two binder systems [21], PARALOID EXL 2314 based on acrylic modifier and LOTADEL AX8090 based on a random terpolymer of ethylene, methyl acrylate, and glycidyl methacrylate, which are commercial impact modifier systems for thermoplastic polyester resins, together with EPIKOTE Resin 05390 were compared under mode I deformation regarding their influence on the mode I fracture toughness. From all the selected binders, the acrylic modifier-based binder PARALOID EXL 2314 showed the best performance in terms of the homogeneity of the pCBT laminate and the magnitude of the final fracture toughness. With 3 wt.%, the process efficiency was improved, but the mode I fracture toughness was decreased from 600 to 400 J/m^2^ compared to the reference laminate. It increased up to 550 J/m^2^ when a further 7 wt.% of the binder was added. However, the mode II fracture toughness of composite material with 3–7 wt.% of such binder was improved from 27 J/m^2^ by 124.4–151.6%. Moreover, 1–3 wt.% of this binder significantly improved flexural properties to 650–831 MPa. The epoxy binder showed rather similar impact on the mode I fracture toughness but mode II fracture toughness was not investigated further. The LOTADEL AX8090 showed decreased mode I fracture toughness compared to reference laminate. Other properties with such binder were not shown.

It can be concluded that due to the increased requirements for modern materials and, as a consequence, the need to use new types of matrices, new binder systems have to be developed for the LCM technology, which, accordingly, will lead to a significant increase in research in this field in the coming years.

## 5. Influence of Binders on LCM Technology

Binders have a significant impact on the manufacture process of composite materials, namely on the preforming process, preforms’ quality, their permeability, and on the curing pattern of the matrix. First of all, they change the properties of the preforms. For example, the researched consolidation and relaxation behavior of stacks of continuous strand random glass mat with a thermoplastic polyester binder VetrotexUnifilo U-750 of known flow characteristics showed glass fiber tow flattening at the greatest rate at high temperatures (135–177 °C) and at low closing speeds (0.02 mm/s) [79]. Flattening of the fiber tows introduces small gaps along which the binder flowed mainly due to the squeezing force; capillary forces played a minor role there. The redistribution of binder facilitates further compaction. The extent of binder redistribution was also governed by the binder viscosity characteristics. The reduced binder viscosity at high shear rate at the highest closing speed or with increased temperature led to a reduction in pressure necessary to obtain a given fiber volume fraction. Above the binder melting point, some relaxation of the mats was observed because fiber tows were more able to move about and thus to springback when the pressure was released.

V. Rohatgi and L.J. Lee showed that preform springback also occurred when the force exerted by the compressed woven graphite fibers, upon release of an externally applied load, was greater than the “holding” force provided by reactive (bismaleimide, 100 µm size powder based on 4,4′-bismaleimidodiphenylmethane and diallylbisphenol A) or non-reactive (polymethylmethacrylate, Mw = 250,000, powder of the same size) binders [16]. Increasing the degree of cure of the binder or increasing its concentration reduced the amount of springback in U-shape bending of fiber preforms. But it was important to avoid gelation of the binder, because, ideally, the binder should be soluble in the incoming matrix resin.

Furthermore, it was found that the “holding” force depends upon the binder modulus and the wetted surface area of the preform. The surface area of the preform covered by the binder was either inter or intralayer, depending upon the rheological properties of the binder and the preforming conditions. During the preforming stage the binder can melt, coagulate, and flow along the capillaries blocking the pore spaces within the preform, which may affect fiber wetting by lowering the capillary pressure and may hinder resin impregnation into fiber tows. For the same binder concentration, better springback control was achieved when binder was inside the fiber tows. Moreover, since mold filling is governed primarily by resin flow between the fiber tows, permeability or flow resistance was not affected much by intra-layer coverage of the binder.

The compaction behavior of bindered textile preforms has been significantly influenced due to the presence of powder epoxy binder (EPIKOTE resin 05390), which was investigated by the Taguchi method [80]. Instead of the commonly applied three parameter model that correlates the fiber volume content and the compaction pressure during compaction of textile preforms under 0.1 MPa, a modified four parameter model was proposed to extend the modeling range up to 0.9 MPa, which gave more accurate prediction of compaction response of the bindered textile preforms. The optimal compaction and preforming parameters settings for fiber volume fraction and residual preform thickness were compaction temperature 190 °C, binder activation temperature 90 °C, binder content 3%, and binder activation time 0.5 min [81]. The compaction temperature was the main factor that had the highest importance on the fiber volume fraction. Thus, with the increase in temperature from 25 to 190 °C the fiber volume fraction increased from 67 to 73% at the compaction pressure of 0.3 MPa. The residual preform thickness was also affected by binder activation time (the estimated contribution was 1.32%), binder content (7.32%), and binder activation temperature (23.24%).

Preforming experiments showed that with the increasing weight fraction of binder (Germany BUF008) [82], the initial thickness of carbon fiber preform increases, as well as the relaxation rate, the compression distance, and preforming time. As the weight fraction of binder increases, the proportion of elastic recovery becomes smaller, while the proportion of viscoelastic recovery increases. The binder content of 4 wt.% was a relative optimal value for preforming process. The compression thickness can be reduced by lowering the compression speed. Then, the volume fraction of the fabrics can be raised, but the preforming time will be increased too. Under different maximum pressure, for fabrics with and without binder, the recovery thickness increases with increasing maximum pressure. Besides, as the holding time increases, the permanent recovery ratio increases.

A unified viscoelastic model to predict the compression, recovery, and relaxation behaviors of carbon fiber fabrics with binder, consisting of parallel Maxwell and non-linear spring elements, was proposed [83,84,85]. The correlation factors for all contents of binder (0–12 wt.%) were larger than 0.9 for compression, and were larger than 0.987 for relaxation. A viscoelastic model with correlation factors larger than 0.997, consisting of parallel dashpot and spring elements was developed to predict the viscoelastic responses in recovery stage. In the compression stage, the elastic stress is the main stress, and the viscoelastic stress is very small. Elastic modulus increases non-linearly and rapidly with the loading time. As the content of the binder increases, the elastic modulus gradually decreases due to the formed binder layer. For the relaxation stage, the total stress relaxation ratio is improved, while the elastic stress relaxation ratio is reduced, indicating the addition of the binder can significantly improve the relaxation ability. It was confirmed that the increasing content of the binder enlarges the relaxation time.

The mechanical behavior of bindered (EPIKOTE Resin 0539) and dry woven fabrics (SIGRATEX C W305-PL1/1), which has been examined under consideration of the forming temperature [86], showed, that binder quantity and forming temperature have a substantial impact on the in-plane shear behavior. When temperatures dropped, the impact of the binder and activation time increased. At higher temperatures >70 °C the binder did not provoke any substantial changes of the bending behavior whereas at ambient temperature, the binder led to augmented bending stiffness. In static and dynamic friction between the textile layers, the binder’s influence highly depended on activation time. The impact of the binder was particularly obvious at lower forming temperatures <70 °C, whereas it may already be neglected at 100 °C. These investigations reveal that temperature control constitutes a major factor for achieving good formability and stability of the preform.

As can be seen from the previous sections, some binders, as well as powdered epoxy binder PRETEX 110 [87], often diminish the permeability of preforms [88]. For the preform containing 4% of binder, the unsaturated permeability value was 35% less than the 3% binder content preform [87]. Therefore, a lot of research is being carried out in this area as well. The main works in this field are summarized in Table 5.

Three different binders based on epoxy powder EPR5390, co-polyamid veil PA1401, and acrylate styrene co-polymer powder Vinnex LL2319 A9133 showed different permeabilities [89]. Permeability of preforms with 2 wt.% epoxy binder was first increased and then decreased with increasing binder content (4–6 wt.%). This happened because at low binder contents new flow channels were created in the vicinity of a binder particle and at higher binder contents they were gradually clogged. Permeability of preform with 2 wt.% of PA1401 veil was increased too, but in lower range. The Vinnex binder decreased the permeability even at low concentration of binder in the preform (2 wt.%). It should be understood that often the permeability of preforms is determined without the use of actual matrices, but with the use of test fluids (usually plant oils) and they can give different results [90], because they have different surface energy and dissolubility of the binder compared to the actual epoxy resin. Furthermore, the noteworthy permeability behavior at high temperatures was found when using RIMR135 resin (DGEBA) as test fluid. The permeability increase followed up by a major decrease over system temperature was observed. This discovery needs further investigation. The use of these binders increased the required peel force from 0.2 N up to 0.7 N and bending modulus from 40 to 200 MPa when up to 7 wt.% of binders were added because a greater number of filaments were bonded at a greater number of individual bonding points. The mean free path between two supports decreases and thus more load is transferred into the fibers and the preform becomes stiffer.

Moreover, it was found that changing the amount (4.04–9.76 wt.%) of bisphenol-A-based powder binder (XB 3366) and binder particle size was easy to implement but had a relatively small impact on the out-of-plane-permeability of preforms manufactured by dry fiber placement (DFP, an automated preforming process) [9]. The positive effect of having more binder particles was partially counteracted by blockage of the resin flow at higher binder content. Optimizing the particle size was preferable in terms of effectivity. The as manufactured mixture of binder particle sizes (25–300 µm) showed the lowest permeability, large-sized binder particles increased it, while medium-sized particles showed the highest permeability. Modification of the layup sequence of the filler (the rovings were placed with a gap of one roving apart) more than doubled the out-of-plane permeability due to the introduction of undulation. Tufting of DFP-preforms showed the highest effectivity with a permeability increase of more than a factor of 30, although this represented an additional step required for an efficient LCM process.

In another work the 5 wt.% of powder reactive epoxy binder Momentive EPS620 [91], cured under vacuum at 120 °C for 10 min, added to a unidirectional carbon fiber non-crimp fabric reduced the preform permeability orientated along the fiber direction by 60.1%, and the 10 wt.% of the binder reduced the permeability by additional 3.7%. The permeability transverse to the fiber direction was less sensitive to an increase in the binder level. In case of DFP preforms when the binder content was increased from 5 wt.% to 10 wt.%, the permeability of the preform at a fiber volume fraction of 31% was reduced by 27%, and at fiber volume fraction of 46% the reduction was only 15%. A permeability model was developed, which accounts for local features of the 3D fiber architecture of carbon DFP preforms, i.e., number of tows, orientation, and through-thickness spacing, for approximation of local permeability values.

A greater effect shows a non-reactive powder type epoxy binder Araldite LT3366 [92]. Both the permeability in the 90° direction and the permeability in the 0° direction decreased by about 80% with binder treatment (without heat treatment during the preforming process) at fiber volume fractions of 55% and 60%. In the case of the permeability in the out-of-plane direction with heat treatment during the preforming process it decreased by approximately 98%. In particular, in this case the binder was flattened and impregnated within the fibers during the preforming process, as was observed through micro-optical observation, which hindered the flow in the out-of-plane direction.

Additionally, some of the first works related to the binders, carried out in the academic environment, were associated with the determination of their influence on the formation of composite materials by LCM technologies. The experimental work and an analytical description for mass transfer of soluble in the polyester resin low molecular weight thermoplastic polyester binder from a preform to the resin during non-isothermal impregnation by LCM methods showed that such binder allows faster wet-out of the preform [37].

Binder applied to the mat in an amount of 4–10 wt.% may dissolve unevenly in the resin near the inlet of the impregnation system and near the other end, which can lead to a distribution of properties, such as stiffness, along the product. Uneven shrinkage, as well as different reaction rates and exotherm, which are also influenced by thermoplastic, can lead to different stresses in the matrix, and therefore it is important to be able to predict the distribution of the binder in the resin part during the impregnation process. Moreover, binder dissolution increases the resin viscosity, which significantly affects the process of impregnation of the preform, since it requires a higher injection pressure for the same injection rate, which will hinder wet-out and generate inhomogeneities. Finally, increasing viscosity or decreasing mobility along the injection flow direction favors the development of “fingering” flow instabilities and inhomogeneities in the material. The rate of binder dissolution and the binder concentration profile obtained in the filled mold under a variety of conditions, as well as the binder washout curves from glass mats were determined, and a mathematical model for the mass transfer of binder from the preform to the resin was developed. Combined with a viscosity model, it is able to predict the resin viscosity variation in the mold.

The investigation of the effect of the reactive HP03 binder powder on the chemorheology of an RTM6 epoxy resin showed [93], that the autocatalytic cure model by Karkanas and Partridge can be successfully applied to describe the cure kinetics of the obtained epoxy systems, and a modified version of the Williamse-Landele-Ferry equation that took into account the gelation and the effects of crosslinking can be used as a chemorheological model. The DSC and rheological results prove that the addition of the binder generally causes a significant change in the kinetics of the cure reaction. The shift to lower temperatures of the beginning of the crosslinking reaction, the heat flow peak, and the dynamic gel temperature can be observed. Furthermore, the addition of preforming binder leads to a significant reduction of the processing time of the resin matrix (Gel Point in isothermal conditions at 180 °C reduces from 30 to 17 min).

Based on the above, we can conclude that when using binders in LCM technology, modeling (both theoretical and using an array of experimental data) of the processes occurring during the manufacture of composites is of particular importance for obtaining a homogeneous defect-free material.

## 6. Automatic Preform Layout Using Binders

From the very beginning of the production of composite materials, the reduction of their manufacture cost was one of the main areas of research [94]. To achieve this goal there are three main approaches: (1) To produce components molded to net thickness so as to reduce assembly fixture cost and labor time; (2) to replace pre-preg with a lower cost form of material; (3) to use approaches which enable the automation of material deposition at a much higher rate than current machines. LCM technologies satisfy the second approach, and the use of binders and automatic methods for assembling preforms can help in the implementation of the third approach to reduce cost, because the hand laminating is very time intensive and requires skilled labor. That is why these methods found use in the production of aviation structures [26].

The function of one of the first methods of automated deposition of the bindered tapes was to place in parallel closely edge butted strips of tape in successive layers, consolidated into a robust wrinkle free preform [95]. This approach was titled bindered tape placement (BTP). Cost modeling of the manufacture of a range of sizes of skin components by the BTP and conventional pre-preg tape laying approaches showed a reduction of 52% for a regional aircraft aileron skin and 60% for a regional aircraft tailplane skin. When using this system there were two major problems: the lack of through thickness permeability and high possibility of damage of the underlying layers during laying [22]. With binder supporting the tape at 10 mm intervals, the tape applied to the preform was unadhered at each cut end. This caused successive layers to catch and damage the underlying layer. Two alternatives based on UniweaveConstructex with two types of epoxy resin binder (one as a solvent diluted spray and the other as a sprinkled powder) were proposed to eliminate these problems. A spray binder was applied on line from the tape-laying head, which was the initial choice for application cost reasons. Proposed rubber-type binders that provided a dry, tacky finish, tapes suitable for adhering the preform were not completely compatible with the matrix resin and hence could not be qualified for use in aircraft. A compatible epoxy resin could not be dried fast enough to prevent coating of the head rollers which became adhered to the passing tape. The solution to the problem was to precoat the tape off-line with an epoxy powder binder, CYTEC 790. This was a relatively costly step, being a separate, additional process, but produces a stiff tape with 1.25 wt.% of powder on each side. At the preforming temperature of 80 °C, the powder became tacky and adhered the preform together when compressed by the deposition rollers and more thoroughly when using a hot table vacuum bag debulking system at a later stage. After cooling, the preforms were rigid and compacted, which allowed them to be trimmed accurately to size. This modified technique was used to produce large, net shape, net thickness preforms ready for processing by both RTM and vacuum infusion techniques.

Subsequently [2,95,96], these technologies were improved and modified in order to achieve the required parameters of preforming process and preforms characteristics, while the binder systems were also adjusted to them in order to obtain the best properties for both the preform, including curved, and the finished product. In a comparative study of various commercial DFP tapes, it was found that thermoplastic binders are also suitable for producing bindered tapes with the desired properties, from which preforms with the required fiber volume fraction can be made [97]. It has been demonstrated that DFP can help to improve the composite quality, resulting in a higher fiber volume fraction up to 56.3%, a less porosity of 0.60% and a better surface flatness with coefficient in thickness variation of 3.75%, comparing to the values of 47.80% in fiber volume fraction, 2% of porosity and coefficient of thickness variation of 8.11% for manually lay-up laminates [98].

Because of all this, this technology is increasingly used today, and new studies appeared recently where it was adapted to new topics, for example, for obtaining products from recycled carbon fibers or carbon/steel fiber hybrid preforms [99,100].

It should be noted that in spite of the higher productivity and low production cost when using automatic laying out in comparison with manual laying, automatic laying out begins to justify itself only with a significant scale of production. This is due to the need to develop special equipment, since it is not universal and is intended for certain products, fillers, and binders. In addition, for structurally complex products, automated equipment will be even more expensive.

## 7. Conclusions

Based on this work, it can be concluded that modern technologies for the production of composite materials are often based on the use of various binder systems. These technologies allow you to quickly, cheaply, and efficiently obtain complex-profile products used in many branches of science and technology, such as the manufactures of aircrafts, cars, yachts, and devices for household purposes. However, as follows from the analysis of the literature, such systems require a more thorough study, since in many research there is no extensive amount of data required for a complete assessment of the effect of a binder on the properties of the resulting preforms, their permeability, infusion parameters, and resins curing patterns, as well as on the operational properties of the obtained materials.

The most widespread, undoubtedly, are binders based on epoxy and acrylic resins, which is due to their relative cheapness, the ability to regulate their chemical and physical properties, as well as good qualities of the products obtained on their basis. However, in some cases, special purpose binders can be used, for example, based on polyurethane, polycarbonate, polyamide, and many others.

Due to the possibility of automation, optimization, and versatility of binder technologies, they will undoubtedly develop and be more and more introduced into production.

It should be noted that the rejection of expensive synthetic fillers, the environmental aspects of production, an increase in plant and animal waste contribute to an increase in interest in bio materials. Modern developments in the field of bio-composites are devoted to the production of materials both based on bio-fillers [101] and based on bio-matrices [102]. At the same time, due to the specifics of the feedstock and the characteristics of bio-composites [103], as well as the need for cheap production, most attention is paid to modern LCM processes. Accordingly, one should expect that considerable attention of scientists and engineers will be given to the development of new binders for the production of bio-composites.

## Figures and Tables

**Table 1:** Overview of binders for polyester and vinyl ester resins proposed in the literatures.

Binder	Description	Application and Preforming Conditions	Reference
Isophthalic polyester resins	Powder, 50 μm, dissolve in styrene for more than 10 min, can be mixed with benzoyl peroxide	1 g of powdered resin was sprinkled uniformly over the 5 cm glass mat and water was sprinkled on the mat for improving the retention. This process was repeated 4 times and then mat was heated for 20 min at 200 °C.	[29]
Unsaturated polyester resin	Powder, copolymer of bis(β-hydroxyethyl)terephthalate with maleic anhydride or 2,2-bis(4-hydroxyphenyl)propane, melts at 110 °C, dissolve in styrene for more than 10 min	2.5 wt.% or more of the binder was applied on the mat, water was sprinkled and then the resin was melted at 180–240 °C for 2–3 min.	[31]
UV-curable monomers	Monomers with two types of photoinitiators (partly curable at visible light and at UV light)	1–12 wt.% binder was applied by spraying (or by calendaring) on the glass fiber, and then visible light used to partly cure the binder for subsequent handling. UV light was used for preforming.	[32]
VR-60	Bisphenol A solid vinyl ester, melts at 80 °C, Mw 1950, viscosity at 90 °C 500 Pa·s	2 wt.% of powder was applied to the fibers and then IR heater heat them to 110 °C for 10 min. The preform can be laminated with a press heated to 100 °C at 0.1 MPa for 5 min.	[33]
PD-3402	Powdered acrylic resin with epoxy and unsaturated groups, 80 μm, melts at 130 °C, Tg 45–50 °C, viscosity at 140 °C 600 Pa·s	Same procedure can be used with the heater and press temperature about 150 °C.	[33]
Orgasol 1002	Powdered polyamide, 15 μm, viscosity at 225 °C 200 Pa·s	Same procedure can be used with the heater and press temperature about 230 °C.	[33]
ATLAC 363E,	thermoplastic polyester with fumarate groups, melts at 60 °C, Tg 47 °C	3–9 wt.% of binder was uniformly spreading on the glass fabrics and were heated at 65 °C for 10 min. Preforms were consolidated under 2.5 kPa or more and 80 °C for about 45 min.	[34,35,36,37]
Cured PRETEX 110	Epoxy resin catalyzed with dicyandiamide, melts at 60 °C, reacts at 75 °C, cured Tg 110 °C	Same procedure can be used. Preforms were consolidated under 110 °C for 2 min. Preforms with uncured binder were consolidated under 65 °C for 30 min.	[36]

**Table 2:** Influence of binders on the properties of preforms and composite materials based on the polyester and vinyl ester resins: σ*_f_*– flexural strength; E*_f_*—flexural modulus, G_IC_in—Mode I initiation interlaminar fracture toughness, G_IC_prop—Mode I propagation interlaminar fracture toughness.

Binder	Resin	Fabrics	Binder Conc. wt.%	Peel Strength ^1^, N/cm	σ*_f_* ^1^, MPa	E*_f_* ^1^, GPa	G_IC_in ^1^, J/m^2^	G_IC_prop ^1^, J/m^2^	ILSS ^1^, MPa	Ref.
VR-60, 50 μm	vinyl ester resin based on Epikote 828	HTA-3K E30 200 g/m^2^	0	0	980	54				[33] ^2^
			0.05	10	970	54				
			1	80	990	54				
			2	100	980	54				
			18	350	960	54				
VR-60, 400 μm			2	150	980	54				
			25	450	800	53				
PD-3402, 80 μm			2	150	970	54				
Orgasol 1002, 15 μm			2	45	820	53				
ATLAC 363E	Neoxil 266 polyester	E-glass from CamElyaf	0		330 ± 40	12.4 ± 2.7		720	31 ± 3	[34,35]
			3	0.13 ± 0.005	280 ± 45	13.4 ± 1.3		432	30 ± 5	
			6	0.15 ± 0.005	270 ± 60	16.9 ± 0.6		235	31 ± 3	
	DERAKANE 411-C-50 vinyl ester resin	E-glass Vetrotex 324 0°/90° woven roving	0				576 ± 142	855 ± 152	46 ± 2	[36]
			3	0.65 ± 0.19			455 ± 96	560 ± 140	44 ± 1	
			6	1.21 ± 0.35			291 ± 75	404 ± 112	40 ± 2	
			9	1.64 ± 0.53			273 ± 60	357 ± 60	36 ± 2	
Cured PRETEX 110	DERAKANE 411-C-50	E-glass Vetrotex 324 0°/90°	3	7.85 ± 2.61			928 ± 105	1126 ± 110	45 ± 2	[36]
			6	15.52 ± 3.87			1096 ± 154	1261 ± 177	41 ± 2	
			9	24.50 ± 2.96			1033 ± 114	1257 ± 169	39 ± 3	

^1^ Peel strength, flexural strength, compression strength, Mode I interlaminar fracture toughness and interlaminar shear strength (ILSS) was measured according to ASTM D1876, ASTM D 790, ASTM D 695-M, ASTM D 5528, and ASTM 2344, respectively. ^2^ Peel strength in N/m^2^ and flexural strength was measures according to JIS K 6854-1 and JIS K 7074, respectively.

**Table 3:** Overview of binders for epoxy resins proposed in the literatures.

Binder Nature	Example	Application and Preforming Conditions	Reference
Acrylonitrile-vinyl chloride copolymer	40% acrylonitrile–60% vinyl chloride copolymeric fibers	4 wt.% of the binder fibers was mixed with chopped glass fibers for 60 min and heated for 2 min at 165 °C and 13.8 kPa.	[30]
Non-catalyzed epoxy resins	EPON^®^ Research Resin RSS-1630–semi-crystalline DGEBA	Applying can be done at temperatures higher than 60–70 °C.	[1]
	D.E.R.-662 –solid DGEBA, melts at 55–60 °C	1–15 wt.% of binder powder or its aqueous dispersion was applied to each layer by sprinkling or spraying and assembled together at 80–110 °C for 30 min. The preforming was done at 95 °C for 30 min.	[41,42]
Catalyzed epoxy	PS500–powdered version of PR500 fluorene epoxy resin	1–40 wt.% of the binder was applied with the electrostatic powder fusion coating method.	[43]
	PT500–powdered version of PR500 epoxy, melts at 60–80 °C, 40 μm, cured Tg about 190–250 °C	3–11% of binder was uniformly applied to one surface of fabric, and heated by an oven or under an IR lamp at 100 °C or at 160 °C for 1 min to obtain the fabric with the binder outside or inside the fiber tows, respectively.	[13,44]
Liquid catalyzed RTM epoxy	RTM6	A thin layer of resin on the fabric was obtained with spray nozzle	[45]
	Prime 20LV	A pointwise binder 3D printing was used to apply binder in specific spots.	[12]
Epoxy resins with thermoplastics	67% Epon 1007F (high Mw epoxy DGEBA), 33% Orgasol 1002 D Nat (polyamide, 22 µm), diluted in acetone	3.8 wt.% of binder was applied with a spray gun (20 kPa, 1.2 mm nozzle) and was heated to 130 °C for 20 min. The preforming was done for 20 min at 120 °C and 100 kPa.	[20]
	67% PT500 33% Orgasol 1002 D Nat–powder binder	3.8 wt.% of binder was applied manually and was heated to 130 °C for 5 min. Same preforming process can be used.	[20]
	Solutions of polysulphones or phenoxies in epoxy resins in the form of water emulsions or powders	Binders were applied using dip-coating method and were dried for 3 min at 100 °C and for further 4 min at 130 °C. Fabrics were assembled at 90–100 °C with pressure about 10 N.	[46,47,48]
Thermoplastics	Polyamides with dicyclohexylmethane unit and toluenesulfonamide which have reduced Tg of 140 °C	Binder was applied by an embossed roll and a doctor blade while being allowed to naturally fall on one surface of the fabric, and then it was heated by IR to 160 °C. Preforming temperature was the same.	[49]
	powdered thermoplastics polyesters, including analogues of ATLAC binder	Up to 9 wt.% of the binder was applied on one side of the fabric and heated to 80 °C for 30 min. Preform consolidation can be done at 80 °C for 30 min under vacuum in a vacuum bag.	[19,50,51]
	different thermoplastics in the form of powders, fibers, veils or thin films and their combinations.	The binders can be applied on one or two surfaces of the fabrics with different methods and heated above their melting or softening point.	[52,53,54,55,56,57,58,59,60,61,62]
Cured and uncured epoxies with multiwall carbon nanotubes	An aqueous dispersion of a solid DGEBA epoxy with a 1 wt.% dispersion of NC7000 industrial grade multiwall carbon nanotubes.	The fabric was coated with 6.5–6.7 wt.% of binder using a Kcontrol spreading coater and were subsequently dried overnight in an oven at 50 °C.	[63]
Graphene oxide	Graphene oxide and partially reduced graphene oxide	0.0003 wt.% can be embedded to the surfaces of carbon fabric by anodic electrophoretic deposition process	[64,65]

**Table 4:** Overview of the influence of binders on the toughness of composite materials based on epoxy resins: CAI—compression after impact, G_IC_—Mode I fracture toughness propagation, ILSS—interlaminar shear stress.

Binder	Resin	Fabric	Binder Conc., wt.%	CAI, MPa	G_IC_, J/m^2^	ILSS, MPa	Ref.
Epon 1007F with 33 wt.% Orgasol 10,002 D Nat	RTM 6	C. Cramer & Co. 6 k carbon fiber satin (style 445), 365 g/m^2^	0		300 ± 20	73.0 ± 2.0	[20]
			3.8		285 ± 10	74.5 ± 3.5	
PT500 with 33 wt.% Orgasol 10,002 D Nat			3.8		270 ± 10	71 ± 4.5	
Epoxy mixture of resins with PES: PEES copolymer	Prism ^®^ EP2400	Unidirectional non-crimp fabric, 200 g/m^2^ (Saertex)	0			81	[47]
			4			92.9	
Thermoplastic polyester powder, 250 µm	SC-15	OCF 463 S2-glass fibers	0		1600 ± 176	41.1 ± 1.8	[19]
			2.6		617 ± 193	30.9 ± 4.3	
Poly(4,4′-dipropoxy-2,2′-diphenylpropane fumarate), analogue of ATLAC 363E, 50–250 µm	EPIKOTE MGS RIM 135	E-glass UDO ES500	0		610 ± 60		[50,51]
			1		740 ± 20		
			3		960 ± 150		
			4		930 ± 60		
PA1008 polyamide veil, melts at 100–115 °C	TV-15	T700	0	133			[52]
			4.30	192			
			6.45	273			
PE2900 polyester veil, melts at 115–123 °C			4.30	108			
			6.45	95			
VI6010 ternary elastomer vwil, melts at 105–115 °C			4.30	168			
			6.45	203			
PAX000229A polyamide veil, melts at 127 °C			4.30	214		47.6	
			6.45	230		47.3	
PA1300 polyamide veil, melts at 127 °C			4.30	219		46.4	
			6.45	219		48.1	
PAX030617 polyamide veil, melts at 127 °C			6.45	280		53.1	
PKHP200phenoxy veil	RTM 6	Tenax HTS 12 k	0	195 ± 15	340 ± 20	75 ± 3	[53]
			5	190 ± 7	740 ± 100	74 ± 3	
PA1541 polyamide veil			7	205 ± 5	1200 ± 100	68 ± 3	
Mesh thermoplastic layer	NA	carbon fabrics from Tiangong University, 320 g/m^2^	0			48.1 ± 2.1	[57]
			2.08			50.6 ± 3.3	
			6.25			43.7 ± 2.0	
			8.33			51.5 ± 2.5	
			10.42			38.0 ± 3.4	
CoPA 130 powder	RTM6	Toho J IMS60 E13	0	155 ± 15			[58]
			2	250 ± 10			
			4	270 ± 25			
			6	275 ± 20			
CoPA 180 powder			2	225 ± 10			
			4	260 ± 20			
			6	285 ± 20			
Phenoxy 85 powder			6	245 ± 20			
Phenoxy 95 powder			6	240 ± 15			

**Table 5:** Influence of different binders on the permeability of different fabrics.

Binder	Fabric and Media	Binder Conc. wt.%	Preform Permeability K_x_, ×10^−10^ m^2^	Ref.
PT 500 epoxy powder treated at 80 °C 1 h	AS4-6k, 5 harness carbon fabric; diphenyl-octyl-pthalate oil	0	1.00 ± 0.20	[44] ^1^
		5	0.94 ± 0.06	
		10	0.48 ± 0.08	
		15	0.28 ± 0.08	
PT 500 treated at 160 °C 20 min		5	1.72 ± 0.16	
		10	2.72 ± 0.16	
		15	3.70 ± 0.16	
Thermoplastic film, thickness of 0.05 mm treated at 0.1 MPa	Carbon fabric T700, (Jiangsu Yitai Carbon Fiber Weaving Co); Araldite LY 1564SP with Hardener XB 3486	0	0.25	[60]
		na	0.14	
Same film, treated at 0.6 MPa		na	0.19	
Graphene oxide	TR-30 carbon woven fabric; silicone oil	0	1.65 ± 0.09	[64]
		0.0003	1.68 ± 0.06	
Polymer binder		5	0.56 ± 0.13	
Powdered epoxy resin PRETEX 110, 230 µm	Vetrotex glass woven roving 324, 816 g/m^2^; corn syrup	0	3.5	[87]
		2	2.0	
		3	2.0	
		4	1.3	
Epoxy powder resin Araldite LT 3366 BD	Carbon NCF 50k HPT 610 C090, 0/90; epoxy XB 3585 with Aradur 3475	0	0.4	[88]
		2.5	0.8	
Momentive EPR5390, epoxy powder, Tg 61 °C, 106 μm	S37CX000, carbon, 0/90° biaxial, NCF, 308 g/m^2^; vegetable oil, 0.1 Pa·s	0	0.27 ± 0.03	[89]
		2	0.45 ± 0.04	
		4	0.42 ± 0.05	
		6	0.36 ± 0.07	
PA1401 co-polyamide veil, Tg 95 °C, 6 g/m^2^		2	0.33 ± 0.02	
Vinnex LL2319 A9133 acrylate styrene co-polymer powder, Tg 63 °C, 82 μm		2	0.26 ± 0.01	
Powder epoxy binder Epikote 05390	0/90° biaxial carbon NFC, 557 g/m^2^; plant oil; 75 °C	0	0.53 ± 0.05	[90]
		2.5	0.35 ± 0.08	
	Same fabric, epoxy resin RIMR135; 75 °C	0	0.59 ± 0.08	
		2.5	0.49 ± 0.01	
Reactive epoxy binder Momentive EPS620, cured at 120 °C	UD carbon NCF FCIM356, 375 g/m^2^; engine oil	0	4.04 ± 0.55	[91]
		5	1.61 ± 0.25	
		10	1.55 ± 0.10	
Araldite LT3366 non-reactive epoxy powder binder, softens at above 110 °C, Tg 75–85 °C.	Twill woven carbon fabric T300 (3K); silicone oil KF-96-350cs, 0.340 Pa·s	0	0.73	[92]
		<3	0.15	

^1^ Normalized permeability to the fabric layers without adding any binder.

## Data Availability

The data presented in this study are available on request from the corresponding author.
